# Improving Muscat Hamburg Wine Quality with Innovative Fermentation Strategies Using *Schizosaccharomyces pombe* Derived from Fermented Grains of Sauce-Flavor Baijiu

**DOI:** 10.3390/foods13111648

**Published:** 2024-05-24

**Authors:** Xiaotong Lyu, Yifei Zhou, Furong Li, Meiyi Zhou, Chunhui Wei, Liangcai Lin, Xin Li, Cuiying Zhang

**Affiliations:** 1 State Key Laboratory of Food Nutrition and Safety, Key Laboratory of Industrial Fermentation Microbiology, Ministry of Education, Tianjin Key Laboratory of Industrial Microbiology, College of Biotechnology, Tianjin University of Science and Technology, Tianjin 300457, China; xiaotonglv@tust.edu.cn (X.L.); zhouyifei1175@163.com (Y.Z.); 18984938259@163.com (M.Z.); lclin@tust.edu.cn (L.L.); lixin2021@tust.edu.cn (X.L.); 2 Guizhou Guotai Liquor Group Co., Ltd., Renhuai 564500, China; lifr9294@163.com; 3 Liquor Making Biological Technology and Application of Key Laboratory of Sichuan Province, Yibin 643000, China; wei_chunhui@suse.edu.cn

**Keywords:** *Schizosaccharomyces pombe*, *Saccharomyces uvarum*, non-*Saccharomyces* yeast, *Torulaspora delbrueckii*, Muscat Hamburg wine

## Abstract

This study investigates innovative approaches to improve the quality and aroma characteristics of Muscat Hamburg wine production by substituting the conventional *Saccharomyces cerevisiae* yeast with an efficient fermentation strain of *Schizosaccharomyces pombe*. The typical use of *S. cerevisiae* in Muscat Hamburg wine often leads to uniformity and prolonged processing times, requiring subsequent malolactic fermentation to degrade excessive malic acid. The study advocates for the replacement of *S. cerevisiae* with a specific *S. pombe* strain, *Sp-410*, isolated from the fermented grains of sauce-flavor Baijiu, a Chinese spirit. Muscat Hamburg wine fermented with the *S. pombe* strain demonstrates decreased malic acid levels, offering a potential alternative to malolactic fermentation. However, exclusive *S. pombe* fermentation may result in an overproduction of acetic acid metabolites, leading to a monotonous taste. In response, the study proposes a mixed fermentation approach, combining the *S. pombe* strain with a *Saccharomyces uvarum* strain and a non-*Saccharomyces* yeast, *Torulaspora delbrueckii*. The optimized mixed fermentation strategies (M:SP+TD and M60SP+TD) involve specific proportions and intervals of inoculation, aiming to enhance the quality and aroma complexity of Muscat Hamburg wine. In conclusion, this research contributes to advancing the production of high-quality Muscat Hamburg wines, utilizing *S. pombe* as the primary yeast strain and implementing mixed fermentation methodologies.

## 1. Introduction

The distinct flavor of a wine is achieved through an appropriate equilibrium among sugar (sweetness), organic acids (sourness), and polyphenols (bitterness/astringency) [1], particularly achieving an optimal balance between sugar and acid levels [2]. The primary organic acids present in wine are *L*-tartaric and *L*-malic acid, accounting for 70–90% of the overall grape acidity [3]. The concentration of malic acid in grapes can range from 1 to 10 g/L, with various factors influencing this, the most significant being the prevailing climate [2]. Excess malic acid in wine not only imparts an acidic taste, but also provides a substrate for lactic acid bacteria, which can cause spoilage of the wine after bottling [4]. To guarantee the wine’s physical, biochemical, and microbial stability and quality, it is crucial to eliminate any surplus malic acid from the wine [5]. Biological deacidification is frequently employed among the various approaches to reducing the surplus malic acid of wine [2].

Beneficial microorganisms or natural antimicrobial agents are frequently employed in the production of diverse fermented products to enable better control over the fermentation process, expedite maturation, boost safety, and enrich the flavor [6,7,8,9]. Malolactic fermentation (MLF) and malo-ethanolic fermentation (MEF) are two methods for achieving biological deacidification [2]. Malolactic fermentation (MLF) refers to a biological process where tricarboxylic *L*-malic acid is decarboxylated to form dicarboxylic *L*-lactic acid and CO_2_, resulting in deacidification [10]. MLF, when applied in winemaking processes, can impart wine with a milder taste and a smoother mouthfeel, which can also contribute to the flavor and aroma of the final product depending on the grape cultivar and wine style [11]. In the winemaking process, certain lactic acid bacteria (LAB) such as *Oenococcus oeni* can carry out malolactic fermentation (MLF), a favorable change facilitated by the malolactic enzyme. This enzyme is inherent only in some LAB species that are naturally selected during alcoholic fermentation [12]. However, the progress of malolactic fermentation (MLF) during wine fermentation can be affected by various factors, such as glucose and SO_2_ concentration, pH, fermentation temperature, thiamine, biotin content, and ethanol concentration. Unfortunately, these factors can potentially lead to stuck or sluggish MLF [13].

Malo-ethanolic fermentation (MEF) is another efficient method of biological malic acid deacidification, which is predominantly performed by yeast species like *Schizosaccharomyces pombe* (*S. pombe*) and certain strains of *Saccharomyces*. These yeasts utilize intracellular malic enzymes to convert malic acid into pyruvate [11]. Yeast species like *S. pombe* and *Zygo-saccharomyces bailii* can degrade large amounts of malic acid, whereas yeast species like *Saccharomyces* are expected to be the most inefficient metabolizers in terms of extracellular malic acid degradation [2]. One possible explanation for the poor degradation of malic acid by *Saccharomyces cerevisiae* is that the mitochondria where malic enzymes are located are dysfunctional, and the number of mitochondria decreases under fermentative conditions [14]. In contrast, *S. pombe* can degrade malic acid effectively, since it possesses an intracellular malic enzyme with a very high substrate affinity and the cytosolic location of the malic enzyme allows the yeast to efficiently convert malic acid to ethanol. Therefore, *S. pombe* has an active transport mechanism for absorbing malic acid [2]. 

*S. pombe*, commonly referred to as fission yeast, exhibits significant capabilities in the wine fermentation process. Notably, it diminishes malic acid [15] and gluconic acid [16] and enhances pyruvic acid levels [17], thereby effectively improving the taste profile [18] and color stability of wines [19]. Furthermore, it plays a crucial role in controlling the levels of ethyl carbamate, biogenic amines, and ochratoxin A [20], ensuring the safety of the wine [21]. Most importantly, fission yeast matches the alcoholic fermentation capacity of traditional winemaking yeasts [22] and shows remarkable tolerance in extreme conditions characterized by high alcohol content, osmotic pressure, and SO_2_ levels [17,22,23].

Wine fermentation using only *S. pombe* can result in the production of metabolites that adversely affect wine quality, including excessive acetic acid, hydrogen sulfide, acetaldehyde, ethyl acetate, and acetoin [23]. *S. pombe* was conventionally regarded as a spoilage yeast. However, in the past decade, it has gained acceptance for its distinctive capability to deacidify malic acid, thereby mitigating the intense acidity in wines. This is achieved by converting malic acid to ethanol and CO_2_, without the production of lactic acid, as observed in lactic bacteria. Furthermore, in recent years, *S. pombe* has been employed to address challenges in the modern winemaking industry, specifically pertaining to enhancing food quality and ensuring food safety [24]. The fermentation process associated with these yeasts is complex and expensive [25], thereby restricting its extensive utilization in the wine fermentation industry.

Current research on the utilization of *S. pombe* in winemaking focuses primarily on three key areas. First, researchers are selecting strains of *S. pombe* with superior winemaking characteristics [23]. Second, they are exploring the potential benefits of using *S. pombe* in combination with other yeast species during fermentation [26]. Third, investigations involve employing protoplast fusion and gene cloning techniques to express relevant enzymes in *S. pombe* [27].

This study aimed to investigate the effect of different mixed strain strategies on malic acid degradation in Muscat Hamburg wines (a local specialty red wine in Tianjin, China, with distinctive regional characteristics) by subjecting them to multi-strain mixed fermentation. The *S. pombe* yeast strain *SP410*, specifically selected from the Jiupei of sauce-flavor Baijiu (a Chinese spirit) in our previous research, was utilized for fermenting Muscat Hamburg wine in this study due to its exceptional fermentation kinetics [28]. Additionally, a *Saccharomyces uvarum* strain named *WY-1*, known for its superior fermentation performance in Muscat Hamburg wines, was employed [29]. Simultaneously, four non-*Saccharomyces* cerevisiae yeasts, namely, *Torulaspora delbrueckii* (*TD*), *Pichia kluyveri* (*PK*), *Issatchenkia orientalis* (*IO*), and *Wickerhamomyces anomalus* (*WA*), were utilized to identify the appropriate mixed fermentation strategies for the fermentation of Muscat Hamburg wine. The primary goal of this study was to develop a mixed fermentation method that utilizes *S. pombe* as the main strain, chosen for its unique ability to deacidify malic acid, with a specific focus on fermenting Muscat Hamburg wines. The addition of non-*Saccharomyces* yeast is intended to mitigate the negative effects associated with the sole fermentation of *S. pombe*, thereby enhancing a better overall flavor profile and structural quality of Muscat Hamburg wines. Non-*Saccharomyces* yeast and *S. pombe* complement each other’s strengths and weaknesses, maximizing their combined benefits.

## 2. Materials and Methods

### 2.1. Microbial Strains

The *Schizosaccharomyces pombe* (*S. pombe*, strain *SP410*) employed for this study originated from the Jiupei of sauce-flavor Baijiu (a Chinese spirit) in our previous research. The wild-type industrial *Saccharomyces uvarum* (strain *WY-1*) was selected from Muscat Hamburg grapes procured from Ningxia Province, China. Meanwhile, four non-*Saccharomyces cerevisiae* yeasts, namely, *Torulaspora delbrueckii* (*TD*), *Pichia kluyveri* (*PK*), *Issatchenkia orientalis* (*IO*), and *Wickerhamomyces anomalus* (*WA*), were provided by the Species Laboratory of Tianjin University of Science and Technology.

### 2.2. Alcohol Fermentation

Freshly picked Muscat Hamburg grapes (a local specialty red wine in Tianjin, China, with distinctive regional characteristics) were used to sort, remove impurities, remove stems, and crush, and then the grapes were divided into 500 mL conical flasks and sterilized. After returning to room temperature, 50 mg/L-100 mg/L SO_2_ was added. Then, the crushed grape juices were placed in a refrigerator at 4 °C for about 12 h and the pH was adjusted to 3.4 with tartaric acid.

The selected strains were inoculated into a 100 mL conical flask with 25 mL YPD, fixing the test tube in a shaker at 30 °C to culture at 180 rpm for 12 h. After that, the selected strains were injected into 250 mL of sterile YPD medium stored in 500 mL conical flask at 10% (*v*/*v*) inoculation rate. The conical flask was fixed in a shaker at 30 °C and cultured at 180 rpm for about 10 h to a cell concentration of 1.0 × 10^8^ CFU/mL. Then, the bacterial solutions were further cultured in a 250 mL sterile centrifuge tube, centrifuged at 5000 rpm for 5 min, washed and precipitated twice with sterile saline, and inoculated to the prepared grape juice at a 5% (*v*/*v*) inoculation rate (using a 500 mL conical flask containing 300 mL of liquid). Then, the inoculated grape must was placed at a constant temperature of 25 °C, shaking the flask every 12 h and recording the weight. When the weight of the conical flask used for fermenting wine remains unchanged for three consecutive days, it indicates that the wine has completed the fermentation process and the contents of residual sugar, malic acid, lactic acid, ethanol, glycerol, acetoin, higher alcohols, esters etc., are satisfactory.

In this experiment, four distinct fermentation approaches were employed: (1) Different inoculation ratios: Mixed fermentation with *S. pombe* and *WY-1* at varying ratios of 1000:1, 500:1, 100:1, 50:1 and 1:1. The fermentation temperature was maintained at 25 °C, with an inoculation quantity of 5 × 10^6^ CFU/mL. (2) Different inoculation intervals were employed: *S. pombe* was initially inoculated to grape juice with an inoculation quantity of 5 × 10^6^ CFU/mL; then, an equal amount of *WY-1* was introduced subsequently to grape juice at intervals of 0 h, 24 h, 48 h, 60 h, and 72 h, respectively, for sequential mixed fermentation. The fermentation temperature remained at 25 °C. (3) Different co-fermentation strategies with non-*Saccharomyces* yeasts were used: *S. pombe* was co-cultivated at a 1:1 ratio with *Pichia kluyveri* (PK), *Issatchenkia orientalis* (*IO*), *Torulaspora delbrueckii* (*TD*), and *Wickerhamomyces anomalus* (*WA*) with an inoculation quantity of 5 × 10^6^ CFU/mL. The fermentation temperature was held at 25 °C. (4) Different mixed fermentation strategies were used: The chosen non-*Saccharomyces* yeast, *Torulaspora delbrueckii* (*TD*), was firstly inoculated into grape juice with an inoculation quantity of 5 × 10^6^ CFU/mL. Subsequently, three mixed fermentation scenarios were conducted: (i) M:SP+TD: Simultaneous inoculation with *S. pombe* and *WY-1* at a 500:1 ratio with inoculation quantities of 5 × 10^6^ CFU/mL, respectively; (ii) M60SP+TD: Simultaneous inoculation with *S. pombe* (5 × 10^6^ CFU/mL), and *WY-1* (5 × 10^6^ CFU/mL) was then inoculated to grape juice after 60 h; and (iii) SP+TD: simultaneous inoculation with *S. pombe* as a control group (5 × 10^6^ CFU/mL, equal proportion with *TD*). These experiments were all conducted at 25 °C, with samples being collected every 12 h and stored at −20 °C for analysis. The chromaticity values of the wines were measured prior to freezing the samples.

### 2.3. Analysis of Metabolites

#### 2.3.1. Determination of Physical and Chemical Indicators Such as Organic Acid, Residual Sugar, Glycerol, and Ethanol in Wine

The wine samples were diluted 10 times with 5 mmol/L H_2_SO_4_ and filtered with a 0.22 μm aperture filter membrane. For organic acid determination, the column of HPLC was Bio-Rad Aminex-HPX 87H (Hercules, CA, USA), and the wavelength of UV-detector was 210 nm. The equipment adopted is HPLC 1260 (Agilent Technologies, Santa Clara, CA, USA). The mobile phase was 5 mmol/L H_2_SO_4_ at a flow rate of 0.5 mL/min. The column temperature was 35 °C and the injection volume was 20 μL. For the determinations of residual sugar, glycerol, and ethanol, the column of HPLC was GH0830078H (300 mm × 7.8 mm) and the detector was RID. The mobile phase was 5 mmol/L H_2_SO_4_ at a flow rate of 0.6 mL/min. The detector temperature was 45 °C and the column temperature was 65 °C. The injection volume was 20 μL. The content of these substances can be calculated based on the retention time and standard curves of the samples. Each determination was repeated three times.

#### 2.3.2. Analysis of Higher Alcohols and Esters in Wine

The distilled wine samples were filtered with 0.22 μm pore membrane filters, and the contents of higher alcohols and esters were determined via GC-FID. The column of GC was Agilent 2909N-223 (30 m × 0.32 mm × 0.5 μm). The temperature of injection port was 200 °C and the detector temperature was 200 °C. The flow rates of carrier gas, hydrogen, and air were 2.0 mL/min, 30 mL/min, and 400 mL/min, respectively. The makeup flow rate was 25 mL/min and the split ratio was 5:2. The injection volume was 2 μL. The contents of these substances can be calculated based on the retention times and standard curves of the samples. Each determination was repeated three times.

### 2.4. Determination of Wine Color

The chromaticity value of the wines was determined using a UV-visible spectrophotometer [30]. This method involved taking a 1 mL wine sample and placing it in a 1 cm optical path cuvette. The absorbance values at 420 nm, 520 nm, and 620 nm were then measured. Each determination was repeated three times. The calculation formula used was as follows:

Chromaticity Value = A_420_ + A_520_ + A_620_

### 2.5. Sensory Evaluation of Wine

The sensory evaluation was performed with slight modifications, following the method used by Lyu [31]. The sensory evaluation was conducted by a trained sensory panel, which consisted of 10 postgraduate students studying at the Tianjin University of Science & Technology. Prior to the experiment, a one-month sensory training regimen (30 min per day) was administered. The panel underwent seven training sessions prior to conducting the wine evaluations. The experimental procedure involved pouring 15 mL of the wine sample into a standard 50 mL wine tasting glass. Subsequently, an olfactory evaluation was carried out, and the sample was rated using a structured numerical scale of 7 points, ranging from 0, indicating no odor, to 7, indicating the most intense aroma. The characteristic aromas of the Muscat Hamburg wine were discussed by the panel, and eight attributes were finally selected: color (7 points), sourness (7 points), rose aroma (7 points), banana aroma (7 points), grassy notes (7 points), pear aroma (7 points), almond notes (7 points), and detection of nail polish aroma (7 points). The experiment was replicated three times for robust analysis.

### 2.6. Statistical Analysis

Statistical analysis was conducted using SPSS 23.0 (Chicago, IL, USA). A one-way analysis of variance (ANOVA) was employed to compare different treatments. Radar chart, bar charts, column charts, line charts, and bar and line graphs were generated using Origin 2021 (MicroCal Inc., Northampton, MA, USA). The heat map was created using TBtools 1.1047 (South China Agricultural University, Guangzhou, Guangdong, China).

## 3. Results

### 3.1. Wine Fermentation

In the fermentation process of wine, two main organic acids, *L*-tartaric acid and *L*-malic acid, play a significant role. Among them, malic acid is more easily metabolized and utilized by microorganisms, which can lead to instability in the winemaking process [32]. A small amount of malic acid can bring a fresh and pleasant aroma to the wine and aid in the absorption of amino acids. However, excessive malic acid can result in a strong sour taste that masks the wine’s sweetness, disrupting the flavor balance and reducing the overall quality of the wine product [33]. Previous studies have shown that *S. pombe*, a specific yeast strain, has a significant advantage in terms of degrading malic acid in wine [34]. 

**Figure 1:**
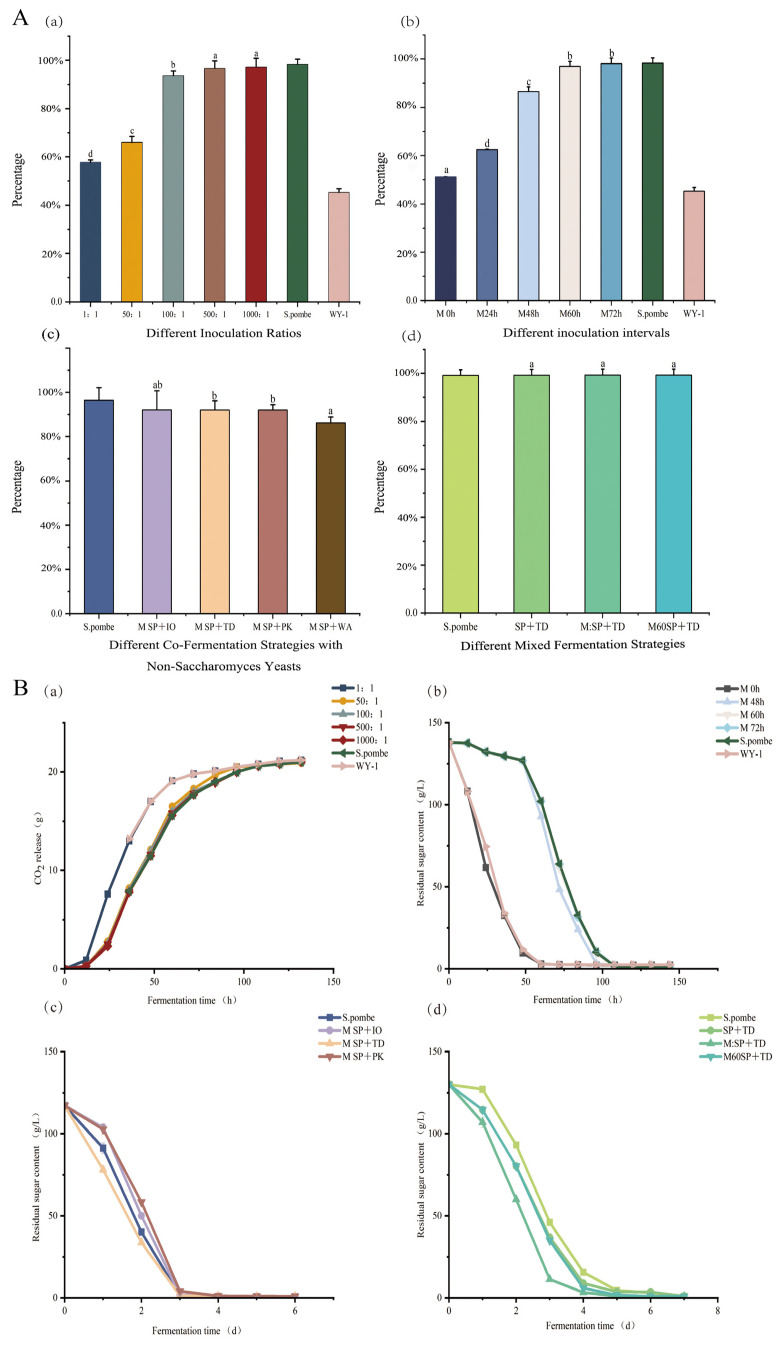
(**A**) Column charts of the degradation rate of malic acid. Averages of triplicate fermentations are shown, with standard deviations as error bars. For each treatment, means with the same statistical letter are not considered significantly different (Tukey, *p* > 0.05). Single-strain fermented wines (*S. pombe* and *WY-1*) were not subjected to statistical analysis alongside mixed-strain fermented wines because the focus of this experiment was to compare different mixed-strain strategies. (**a**) Different inoculation ratios: Co-fermentation was conducted using *S. pombe* and *WY-1* at five different inoculation ratios: 1000:1, 500:1, 100:1, 50:1, and 1:1. In addition, *S. pombe* and *WY-1* were fermented separately as controls. (**b**) Different inoculation intervals: The Muscat Hamburg grape juice was initially inoculated with *S. pombe*, and was subsequently inoculated with yeast *WY-1* (inoculation ratio 1:1) at intervals of 0 h, 24 h, 48 h, 60 h, and 72 h to achieve sequential mixed fermentation. *S. pombe* and *WY-1,* fermented separately, were used as controls for comparison. (**c**) Different co-fermentation strategies with non-*Saccharomyces* yeasts: *S. pombe* was separately co-fermented with *Pichia kluyveri* (PK), *Issatchenkia orientalis* (*IO*), *Torulaspora delbrueckii* (*TD*), and *Wickerhamomyces anomalus* (*WA*) at a ratio of 1:1. Single *S. pombe* fermentation was used as a control. (**d**) Different mixed fermentation strategies: *S. pombe*: Single *S. pombe* fermentation. SP+TD: Co-fermentation of *S. pombe* with *TD* in equal proportions. M:SP+TD: Initial inoculation of *TD* followed by simultaneous co-fermentation of *S. pombe* and *WY-1* at a ratio of 500:1. M60SP+TD: Initial inoculation of *TD* and *S. pombe*, followed by an interval of 60 h before subsequent inoculation of *WY-1*. (**B**) Line charts of fermentation rate: (**a**) Different inoculation ratios, represented by CO_2_ emission. (**b**) Different inoculation intervals, represented by residual sugar levels. (**c**) Different co-fermentation strategies with non-*Saccharomyces* yeasts, represented by residual sugar levels. (**d**) Different mixed fermentation strategies, represented by residual sugar levels.

Figure 1(Aa) shows the results of a simultaneous fermentation experiment using *S. pombe* and *WY-1*, a *Saccharomyces uvarum* strain. The study found that when the proportion of *S. pombe* was higher, the ability to degrade malic acid was stronger. Notably, the mixed fermentation ratio of *S. pombe* and *WY-1* at 500:1 and 1000:1 achieved the highest malic acid degradation rates, reaching 97.25% and 97.47%, respectively. This is because *S. pombe* has an extremely high tolerance to *L*-malic acid and can metabolize it in grape juice to produce pyruvic acid [35]. During anaerobic processes, it is completely metabolized to produce ethanol and CO_2_, while in aerobic processes, it is metabolized to CO_2_ and H_2_O [23]. Figure 1(Ba) presents the results from different inoculation ratios in the fermentation process. It can be observed that all five inoculation ratios completed fermentation without any stagnation. The fermentation rate, represented by the release of CO_2_, was slowest when the inoculation ratios were 500:1 and 1000:1. This indicates that the growth and fermentation rates of *S. pombe* are slow, making it less suitable for wine fermentation alone. However, the addition of the *WY-1* yeast strain can accelerate the fermentation rate, especially when using Muscat Hamburg grapes. In conclusion, when *S. pombe* and *WY-1* are mixed and fermented at a ratio of 500:1, it results in the highest malic acid degradation rate and a relatively high fermentation rate in wine production. The detailed data is contained in the Supplementary Materials.

In Figure 1(Ab), the sequential fermentation of *S. pombe* and *WY-1* at different intervals is displayed. As the interval time increased, the degradation rate of malic acid showed an upward trend. When *S. pombe* and *WY-1* were sequentially fermented with an interval of 60 h or more, more than 97% of the malic acid in Muscat Hamburg wines was degraded. It can be seen in Figure 1(Bb), when *S. pombe* and *WY-1* were simultaneously added to Muscat Hamburg grape juice, the residual sugar consumption rate was the fastest, the same as that of *WY-1* fermentation alone. However, when *WY-1* was added 72 h later than *S. pombe* for sequential mixed fermentation, the residual sugar consumption rate was the lowest, and was essentially the same as that of *S. pombe* fermentation alone (Figure 1(Bb)). This suggests that *WY-1* has a weaker effect on the overall fermentation process when added at an interval of 72 h after *S. pombe*. However, when *S. pombe* and *WY-1* were subjected to sequential mixed fermentation at intervals of 60 and 72 h, the residual sugar contents in the resulting wine were 0.96 g/L and 0.94 g/L, respectively, with no significant difference between them. Therefore, it is speculated that inoculating *WY-1* sequentially with *S. pombe* at intervals of 60 h allows for a highest degradation rate of malic acid with a more thorough fermentation of Muscat Hamburg wines. 

Non-*saccharomyces* yeasts have an influence on the fermentation process of wine to a certain extent. They can produce various fermentation metabolites that can affect the flavor and aroma profile of wine. Some non-*saccharomyces* yeasts are capable of producing volatile phenolic compounds, alcohols, and other aroma compounds, which can contribute unique and desirable flavor characteristics to the wine [36]. However, it is important to note that non-*saccharomyces* yeasts can also have negative effects on wine fermentation, such as excessive amounts of ethanol production. Additionally, certain non-*saccharomyces* yeasts may produce unpleasant odors or off flavors that can detract from the overall quality of the wine [37].

In this experiment, the aim was to enhance the quality of the Muscat Hamburg wines while degrading more malic acid by mixing the *S. pombe* strain with selected non-*saccharomyces* strains. These non-*saccharomyces* strains, namely, *Pichia kluyveri* (*PK*), *Issatchenkia orientalis* (*IO*), *Torulaspora delbrueckii* (*TD*), and *Wickerhamomyces anomalus* (*WA*), have been known to have positive effects on the flavor profile of wine during fermentation [38]. Based on the results shown in Figure 1(Ac), the malic acid degradation ability of different combinations of *S. pombe* and non-*saccharomyces* strains decreased from high to low as follows: *IO* + *S. pombe* (92.87%) > *TD* + *S. pombe* (92.37%) > *PK* + *S. pombe* (92.23%) > WA + *S. pombe* (87.47%). According to the data presented in Figure 1(Bc), during the initial 24 h of fermentation, the residual sugar degradation rates of the mixed fermentation of *S. pombe* and non-*saccharomyces* strains, from fast to slow, were in the following order: *TD* + *S. pombe* (33.40%) > *WA* + *S. pombe* (20.73%) > *PK* + *S. pombe* (12.28%) > *IO* + *S. pombe* (11.72%). Therefore, it can be concluded that the mixed fermentation of *S. pombe* and *TD* displayed the fastest fermentation speed, with a relatively high malic acid degradation rate compared to the other mixed fermentations, which indicates a more efficient fermentation performance.

### 3.2. Volatile Aroma Compounds

In this study, a total of 11 volatile aroma compounds were analyzed in the resulting Muscat Hamburg wines. The primary volatile acid in wine, acetic acid, and its metabolites, including ethyl acetate, isoamyl acetate, acetaldehyde, and ethyl lactate, were examined. However, excessive quantities of acetic acid metabolites can adversely affect the taste of wine [39]. Ethanol and glycerol, the most abundant alcohols in wine, were also investigated as they play important roles in aroma release and taste modulation [40]. Higher alcohols, such as 1-propanol, isoamyl alcohol, isobutanol, and phenylethanol, were assessed as well. These alcohols are metabolic by-products of ethanol and are mainly produced during yeast fermentation. They contribute distinct aroma characteristics, including fruity and floral notes, which enrich the overall flavor profile of wine [41]. The *Saccharomyces cerevisiae* strain *S. pombe* utilized in this research demonstrates a strong capability to degrade malic acid. Additionally, it produces lower levels of acetic acid metabolites compared to *Saccharomyces uvarum* [42].

**Figure 2:**
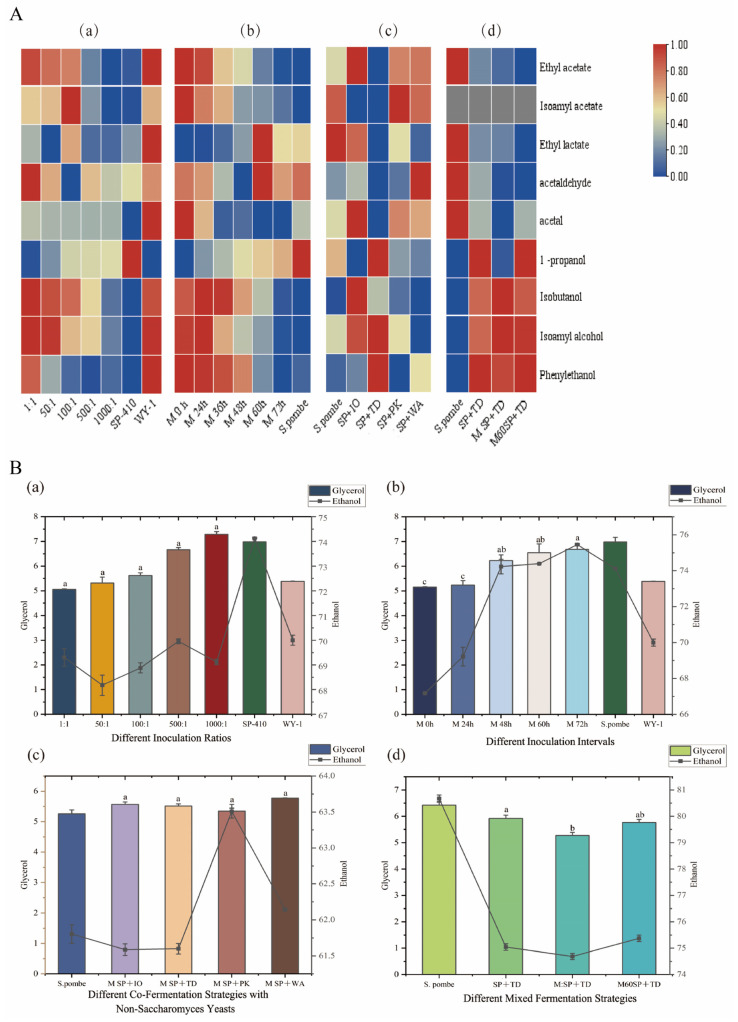
(**A**) Heat map of volatile aroma compounds in the final wines: (**a**) represents different inoculation ratios of *S. pombe* and *WY-1*; (**b**) represents different inoculation intervals of *S. pombe* and *WY-1*; (**c**) represents different co-fermentation strategies with *S. pombe* and other non-*Saccharomyces* yeasts, namely, *Pichia kluyveri* (*PK*), *Issatchenkia orientalis* (*IO*), *Torulaspora delbrueckii* (*TD*), and *Wickerhamomyces anomalus* (*WA*). (**d**) Different mixed fermentation strategies: *S. pombe*: Single *S. pombe* fermentation. SP+TD: Co-fermentation of *S. pombe* with *TD* in equal proportions. M:SP+TD: Initial inoculation of *TD* followed by simultaneous co-fermentation of *S. pombe* and *WY-1* at a ratio of 500:1. M60SP+TD: Initial inoculation of *TD* and *S. pombe*, followed by an interval of 60 h before subsequent inoculation of *WY-1*. The red color in the heat map indicates a higher concentration of the compound in the wines. Conversely, the blue color indicates a lower concentration of the compound. (**B**) Bar and line graphs illustrating the ethanol and glycerol content of different fermentation treatments. The varying colors of the bars signify distinct treatments. Averages of triplicate fermentations are shown, with standard deviations as error bars. For each treatment, means with the same statistical letter are not considered significantly different (Tukey, *p* > 0.05). Single-strain fermented wines (*S. pombe* and *WY-1*) were not subjected to statistical analysis alongside mixed-strain fermented wines because the focus of this experiment was to compare different mixed-strain strategies. (**a**) Different inoculation ratios. (**b**) Different inoculation intervals. (**c**) Different co-fermentation strategies with non-*Saccharomyces* yeasts. (**d**) Different mixed fermentation strategies.

The findings in Figure 2(Aa,Ab) reveal that, as the proportion of *S. pombe* in the fermentation process increases, the levels of acetic acid metabolites decrease gradually. Specifically, when the ratio of *S. pombe* to *WY-1* is 500:1 and the inoculation interval between *S. pombe* and *WY-1* is 60 h, the resulting wine shows relatively lower levels of acetic acid metabolites compared to other treatments, with relatively higher concentrations of ethanol; glycerol (Figure 2(Ba,Bb)); and four higher alcohols: 1-propanol, isobutanol, isoamyl alcohol, and phenylethanol (Figure 2(Aa,Ab)).

The addition of the *TD* strain (Figure 2(Ac)) significantly degrades metabolites like ethyl acetate, isoamyl acetate, and ethyl lactate in wine. However, it is observed that the levels of certain volatile aroma compounds, such as ethanol, glycerol (Figure 2(Bc)), 1-propanol, isoamyl alcohol, isobutanol, and phenylethanol (Figure 2(Ac)), are higher compared to other non-*Saccharomyces* treatments. 

Figure 2(Ad,Bd) demonstrates that there was very little difference in the levels of volatile compounds and glycerol between the two mixed fermentation strategies, M:SP+TD and M60SP+TD. However, there was a significant decrease in the level of acetic acid metabolites in the mixed fermentation (M:SP+TD and M60SP+TD) compared to the single *S. pombe* fermentation (Figure 2(Ad)). This indicates that the mixed fermentation of *S. pombe*, *TD*, and *WY-1* is effective in terms of addressing the issue of an unbalanced wine aroma caused by the higher production of acetic acid metabolites during single *S. pombe* fermentation. The detailed data is contained in the Supplementary Materials.

### 3.3. Wine Color

The chromaticity value of wine is a measurement used to determine the depth and hue of wine’s color, providing insights into the oxidative state and color properties, as well as the age, quality, stability, and potential flavor characteristics of a particular wine [43]. The chromaticity value is determined through optical measurements (UV-Vis spectrophotometer) and analysis of the color composition of wine samples [44]. During the winemaking process, various chemical reactions take place during fermentation that significantly affect the chromaticity value of wine. These reactions involve interactions with pigments and other compounds present in grape skins, as well as the activities of yeast and bacteria [45]. In general terms, wines with higher chromaticity values tend to have deeper and more intense colors, exhibiting rich, vibrant hues, and are often associated with bold and full-bodied characteristics. On the other hand, wines with lower chromaticity values typically exhibit a more delicate and subtle visual appearance [46]. 

**Figure 3:**
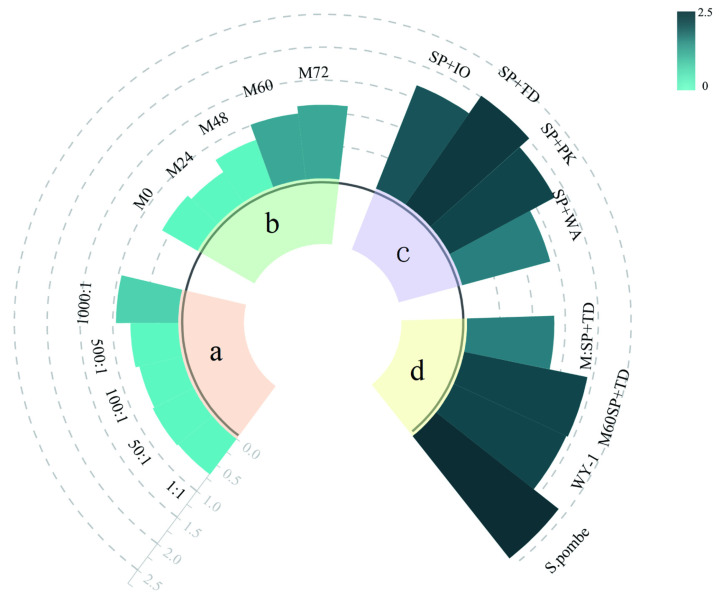
Bar charts of chromaticity values of the final wines: (**a**) represents different inoculation ratios of *S. pombe* and *WY-1*; (**b**) represents different inoculation intervals of *S. pombe* and *WY-1*; (**c**) represents different co-fermentation strategies with *S. pombe* and other non-*Saccharomyces* yeasts, namely, *Pichia kluyveri* (*PK*), *Issatchenkia orientalis* (*IO*), *Torulaspora delbrueckii* (*TD*), and *Wickerhamomyces anomalus* (*WA*). (**d**) Different mixed fermentation strategies: *S. pombe*: Single *S. pombe* fermentation. SP+TD: Co-fermentation of *S. pombe* with *TD* in equal proportions. M:SP+TD: Initial inoculation of *TD* followed by simultaneous co-fermentation of *S. pombe* and *WY-1* at a ratio of 500:1. M60SP+TD: Initial inoculation of *TD* and *S. pombe*, followed by an interval of 60 h before subsequent inoculation of *WY-1*.

As shown in Figure 3a, there was very little difference in the chromaticity values of the wines fermented with different inoculation ratios of *S. pombe* and *WY-1*. In Figure 3b, the chromaticity values for *S. pombe* and *WY-1* at intervals of 60 h and 72 h are reported as 1.01 ± 0.08 and 1.12 ± 0.04, respectively, with no significant difference between them. These values are higher compared to the chromaticity values obtained from other inoculation intervals examined in this study. In Figure 3c, the chromaticity value of the wine co-fermented by *S. pombe* and *TD* is recorded as 2.02 ± 0.03. This value surpasses the instances where other non-*Saccharomyces* yeasts were introduced. This result indicates that the combination of *S. pombe* and *TD* proves to be a favorable combination for achieving a deeper and more intense coloration in this specific type of wine. The results presented in Figure 3d show a significant difference in the chromaticity values between two mixed fermentation strategies. The chromaticity value of M:SP+TD wine was recorded as 1.33 ± 0.01, which is notably lower than the chromaticity value of M60SP+TD wine (1.92 ± 0.03). This suggests that extending the sequential fermentation time interval (to 60 h) between *S. pombe*, *TD*, and *WY-1* could lead to a slightly deeper coloration in the wine. The detailed data is contained in the Supplementary Materials.

### 3.4. Sensory Evaluation

**Figure 4:**
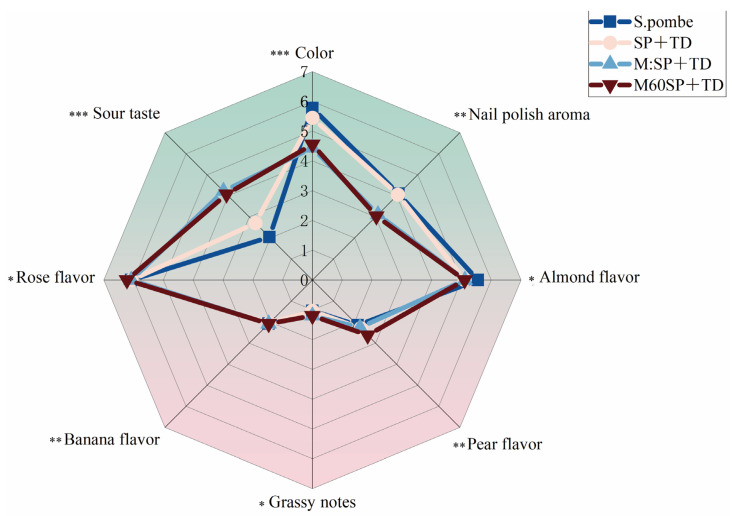
Radar chart of the sensory evaluation of different fermentation methods. (*) *p* < 0.05, (**) *p* < 0.01, (***) *p* < 0.001.

A panel of 10 wine professionals, comprising both teachers and students, was assembled to form the wine evaluation team. The panel’s task was to assess different aspects of the wine samples using a structured numerical scale ranging from 0 to 7. The panel discussed the distinctive characteristics of Muscat Hamburg wine and ultimately selected eight specific aspects: color, sour taste, rose flavor, banana flavor, grassy notes, pear flavor, almond flavor, and nail polish aroma. In Figure 4, the results indicate that significant differences were observed in sour taste, nail polish aroma, and color among the three mixed fermentation strategies (SP+TD, M: SP+TD, and M60SP+TD), while minimal differences were found in the rose flavor, grassy notes, pear flavor, banana flavor, and almond flavor. Firstly, the data indicate that the mixed-fermentation M:SP+TD and M60SP+TD method exhibited a significant stronger sour taste compared to wines fermented using the SP+TD method. Furthermore, the sensory data indicate that wines fermented with SP+TD exhibited a significantly stronger nail polish aroma compared to the wines fermented with M: SP+TD and M60SP+TD. This observation aligns with the instrumental data showing higher levels of ethyl acetate, which is associated with nail polish aroma, as depicted in Figure 2(Ad). Lastly, the sensory data also indicate that the wines produced using the SP+TD method had a significantly deeper color compared to the wines fermented with the M: SP+TD and M60SP+TD methods. This observation is in line with the instrumental data showing higher chromaticity values of the wines measured by a spectrophotometer, as illustrated in Figure 3d. The detailed data is contained in the Supplementary Materials.

### 3.5. Relationship between Instrumental Data and Sensory Data

**Figure 5:**
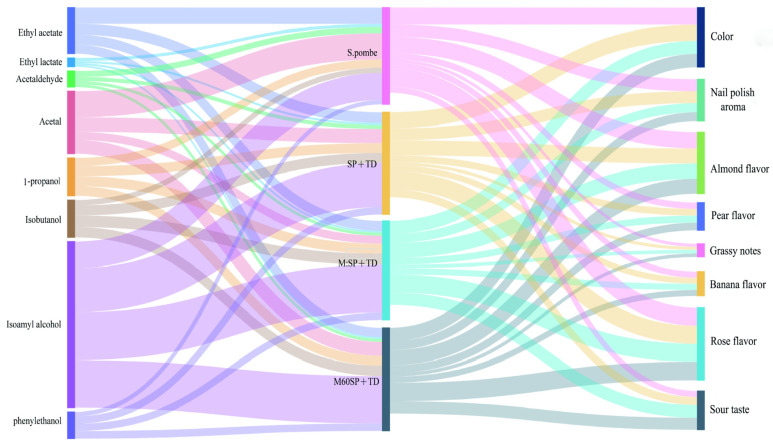
Sankey diagram illustrating the intricate relationships between the volatile aroma compounds and their influence on the overall sensory profile of the wines produced using various mixed fermentation strategies. Each pathway in the diagram would represent a specific aroma compound, and the width of the flow lines would indicate the relative strength or contribution of each compound to the sensory profile. Four mixed fermentation strategies are shown in the Sankey diagram: *S. pombe*: Single *S. pombe* fermentation. SP+TD: Co-fermentation of *S. pombe* with *TD* in equal proportions. M:SP+TD: Initial inoculation of *TD* followed by simultaneous co-fermentation of *S. pombe* and *WY-1* at a ratio of 500:1. M60SP+TD: Initial inoculation of *TD* and *S. pombe*, followed by an interval of 60 h before subsequent inoculation of *WY-1*.

The relationship between instrumental data and sensory data allows for a deeper understanding of the underlying factors influencing the sensory characteristics of a product. By correlating instrumental measurements with sensory evaluations, it is possible to identify key compounds or parameters that contribute to specific sensory perceptions. A Sankey diagram is a visualization tool that effectively presents data flow, revealing the origins, destinations, and quantities involved. Within this diagram, line width correlates with flow quantity, facilitating the identification of significant components in distribution. In the fermentation of sauce-flavor Baijiu, Sankey diagrams vividly depict the sources of various microorganisms throughout the process [47]. Likewise, in light-flavor Baijiu fermentation, Sankey diagrams offer valuable insights into microbial taxa or species and their respective proportions [48,49]. Figure 5 illustrates the relationship between the instrumental data on volatile compounds and sensory evaluation data across various fermentation methods. Graphically, it was observed that the introduction of *TD* and *WY-1* yeast strains in the mixed fermentation with *S. pombe* significantly impacted the composition of the wine. Compared to solely *S. pombe* fermentation, the mixed fermentation resulted in increased levels of 1-propanol, isobutanol, isoamyl alcohol, and phenylethanol. The aroma of 1-propanol is often described as slightly sweet, alcoholic, and solvent-like [50]. Isobutanol, on the other hand, has a characteristic odor that combines alcohol, solvent, and a slightly sweet or fruity note [51]. Isoamyl alcohol is often associated with a scent reminiscent of bananas or other tropical fruits [52]. Lastly, phenylethanol is known for its pleasant and characteristic rose-like floral aroma, with a sweet, rosy, and slightly honeyed scent [53]. The increase in these volatile compounds suggests that the mixed fermentation with *TD* and *WY-1* yeast strains imparted distinctive fruit and flower aromas to the final wines. This finding indicates that the mixed fermentation methods (*S. pombe* with *TD* and *WY-1*) could be a valuable technique for enhancing the aromatic flavor profile of Muscat Hamburg wine.

## 4. Conclusions

Traditional Muscat Hamburg wine production typically involves using commercial *Saccharomyces cerevisiae* yeast for alcoholic fermentation, followed by malolactic fermentation for deacidification. However, this method often results in wines lacking distinct characteristics, as well as those with market homogeneity and lengthy processing times. To overcome these limitations, this study proposes replacing the conventional *Saccharomyces cerevisiae* yeast with a fermentation-efficient strain of *S. pombe*, specifically strain *Sp-410*, which was selected from the Jiupei (fermented grains) of sauce-flavor Baijiu, a Chinese spirit [28].

This study finds that fermenting Muscat Hamburg wine with *S. pombe* lowers malic acid levels, suggesting a potential alternative to malolactic fermentation for deacidification. However, using only *S. pombe* for fermentation may elevate acetic acid metabolites, leading to a monotonous taste and flavor imbalance. To address this, the study proposes using *S. pombe* as the primary strain and combining it with a wild-type industrial *Saccharomyces uvarum* (strain *WY-1*) and non-*Saccharomyces* yeasts for Muscat Hamburg wine fermentation. After comparing various strains and mixed fermentation techniques, the optimal inoculation ratio between *S. pombe* and *WY-1* is determined to be 500:1, with the ideal inoculation interval of *S. pombe* and *WY-1* being 60 h. Furthermore, the most effective complementary non-*Saccharomyces* yeast in conjunction with *S. pombe* is found to be *Torulaspora delbrueckii*, significantly enriching the aromatic profile of Muscat Hamburg wine.

However, this study also has some limitations. For instance, the detection of volatile aroma compounds in the wine is not comprehensive enough, and the impact of different mixed fermentation methods on the typical flavor components of Muscat Hamburg wine has not been considered. Due to uncontrollable factors such as the pandemic, certain parameters, such as volatile acids in the wine post-mixed fermentation, were not tested. In future experiments, there could be new improvements made to the mixed fermentation method.

In summary, the objective of this research was to enhance the quality and aroma characteristics of Muscat Hamburg wines through the utilization of *S. pombe* as the primary yeast strain and as an alternative biological deacidification method, replacing malolactic fermentation. The incorporation of mixed fermentation techniques involving *Saccharomyces uvarum* and *Torulaspora delbrueckii* helps to counteract the potential drawbacks of exclusive *S. pombe* fermentation, ultimately advancing the production of Muscat Hamburg wines of exceptional quality.

## Data Availability

The original contributions presented in the study are included in the article and Supplementary Materials, further inquiries can be directed to the corresponding author.

## Supplementary Materials

The following supporting information can be downloaded at: https://www.mdpi.com/article/10.3390/foods13111648/s1, Table S1: Degradation rate of malic acid. Table S2: CO_2_ release from different inoculation ratios. Table S3: Fermentation rate is expressed by residual sugar consumption. Table S4: Content of metabolic compounds produced at the end of fermentation. Table S5: Ethanol content produced during fermentation. Table S6: Glycerol content produced during fermentation. Table S7: Color values of the finishing wines. Table S8: Descriptive sensory evaluation of the finishing wines.
