# Supplementary material for: Improving Muscat Hamburg Wine Quality with Innovative Fermentation Strategies Using Schizosaccharomyces pombe Derived from Fermented Grains of Sauce-Flavor Baijiu
Source: Foods. 2024 May 24;13(11):1648. doi: 10.3390/foods13111648 (PMC11172094; doi:10.3390/foods13111648)
Supplement: Supplementary file 1 [file foods-13-01648-s001.zip › foods-3005713-Supplementary Materials.pdf]

Improving Muscat Hamburg Wine Quality with Innovative Fermentation Strategies Using Schizosaccharomyces pombe Derived from Fermented Grains of Sauce-Flavor Baijiu

Xiaotong Lyu [1], Yifei Zhou [1], Furong Li [2], Meiyi Zhou [1], Chunhui Wei [3], Liangcai Lin [1], Xin Li [1], Cuiying Zhang [1,*]

[1] State Key Laboratory of Food Nutrition and Safety, Key Laboratory of Industrial Fermentation Microbiology, Ministry of Education, Tianjin Key Laboratory of Industrial Microbiology, College of Biotechnology, Tianjin University of Science and Technology, Tianjin 300457, People's Republic of China; xiaotonglv@tust.edu.cn (X.L.); zhouyifei1175@163.com (Y.Z.); 1656156725@qq.com (M.Z.); lclin@tust.edu.cn (L.L.); lix-in2021@tust.edu.cn (X.L.)

[2] Guizhou Guotai Liquor Group Co., Ltd., Renhuai 564500, China; 782595610@qq.com

[3] Liquor Making Biological Technology and Application of Key Laboratory of Sichuan Province; 147516753@qq.com

[*] Correspondence: cyzhangcy@tust.edu.cn

Table S1
Table S2
Table S3
Table S4
Table S5
Table S6
Table S7
Table S8

Table S1. Degradation rate of malic acid

Different Inoculation Ratios	Malic Acid Degradation Rate	Different Inoculation Interval	Malic Acid Degradation Rate	Different Co-Fermentation Strategies with Non-Saccharomyces	Malic Acid Degradation Rate	Different Mixed Fermentation Strategies	Malic Acid Degradation Rate
1:1	57.7% d	M 0h	51.1% b	SP＋IO	92.0% ab	SP＋TD	99.2% a
50:1	65.9% c	M 24h	62.4% b	SP＋TD	92.0% b	M:SP＋TD	99.3% a
100:1	93.6% b	M 48h	86.5% c	SP＋PK	92.0% b	M60SP＋TD	99.2% a
500:1	96.6% a	M 60h	96.9% d	SP＋WA	86.2% a		
1000:1	97.2% a	M72h	98.1% a				
S.pombe	98.3%	*S.pombe*	98.3%				
WY-1	45.3%	*WY-1*	45.3%				

Table S2. CO_2 release from different inoculation ratios

	Fermentation Time (h)	CO_2 Release (g)	CO_2 Release (g)	CO_2 Release (g)	CO_2 Release (g)	CO_2 Release (g)
		1:1	50:1	100:1	500:1	1000:1
	0	0.00±0.00	0.00±0.00	0.00±0.00	0.00±0.00	0.00±0.00
	12	0.90±0.00	0.30±0.00	0.30±0.00	0.29±0.00	0.28±0.10
	24	7.60±0.10	2.80±0.00	2.60±0.00	2.50±0.00	2.30±0.20
	36	13.00±0.10	8.20±0.10	8.00±0.10	7.80±0.20	7.80±0.10
	48	17.00±0.10	12.10±0.10	12.00±0.10	11.60±0.10	11.50±0.02
**Different Inoculation Ratios**	60	19.10±0.10	16.50±0.10	16.00±0.10	15.80±0.02	15.60±0.10
	72	19.80±0.10	18.30±0.10	18.00±0.02	17.80±0.04	17.70±0.10
	84	20.10±0.10	19.70±0.10	19.00±0.10	18.90±0.20	19.00±0.10
	96	20.50±0.10	20.50±0.02	20.00±0.10	20.00±0.10	20.00±0.10
	108	20.80±0.10	20.60±0.04	20.60±0.10	20.60±0.02	20.60±0.02
	120	21.10±0.10	20.80±0.20	20.80±0.02	20.80±0.04	20.80±0.10
	132	21.20±0.10	20.90±0.10	21.00±0.04	21.10±0.20	21.00±0.10

Table S3. Fermentation rate is expressed by residual sugar consumption

		Residual Sugar Content（g/L）	Residual Sugar Content（g/L）	Residual Sugar Content（g/L）	Residual Sugar Content（g/L）	Residual Sugar Content（g/L）
**Different Inoculation Interval**	**Fermentation time（h）**	**M 0h**	**M 24h**	**M 48h**	**M 60h**	**M 72h**
	0	138.00±0.00	138.00±0.00	138.00±0.00	138.00±0.00	138.00±0.00
	12	108.34±0.20	137.57±0.11	137.57±0.23	137.57±0.20	137.57±0.20
	24	61.70±0.30	132.21±0.20	132.21±0.20	132.21±0.10	132.21±0.10
	36	32.64±0.20	100.93±0.10	129.65±0.10	129.65±0.09	129.65±0.09
	48	9.72±0.20	53.79±0.20	126.92±0.09	126.92±0.20	126.92±0.20
	60	3.03±0.10	29.31±0.10	92.82±0.11	102.09±0.10	102.09±0.10
	72	2.59±0.20	9.49±0.09	48.29±0.20	61.20±0.09	63.87±0.09
	84	2.53±0.10	2.64±0.20	23.91±0.10	31.69±0.01	31.59±0.01
	96	2.31±0.02	2.31±0.11	3.12±0.00	9.16±0.01	10.56±0.01
	108	2.31±0.02	2.30±0.00	2.05±0.00	1.52±0.01	1.72±0.01
	120	2.31±0.02	2.30±0.00	2.05±0.00	0.97±0.01	0.99±0.01
	132	2.31±0.02	2.30±0.00	2.05±0.00	0.97±0.01	0.99±0.01
	144	2.31±0.02	2.30±0.00	2.05±0.00	0.97±0.01	0.94±0.01
**Different Co-Fermentation Strategies with Non-Saccharomyces**	**Fermentation time（d）**	**SP＋IO**	**SP＋TD**	**SP＋PK**	**SP＋WA**	
	0	117.11±0.00	117.11±0.00	117.11±0.00	117.11±0.00	
	1	103.88±2.10	78.14±2.07	102.73±3.07	92.83±0.07	
	2	49.99±0.07	33.71±0.10	58.30±0.10	43.87±0.10	
	3	1.84±0.10	1.40±0.12	4.08±0.12	1.67±0.12	

	4	1.17±0.12	1.08±0.12	1.22±0.12	1.24±0.12
	5	0.89±0.12	0.91±0.01	1.02±0.01	1.13±0.01
	6	0.87±0.01	0.94±0.00	0.94±0.00	1.05±0.00

	**Fermentation time（d）**	**SP＋TD**	**M:SP＋TD**	**M60SP＋TD**
**Different Mixed Fermentation Strategies**	0	114.57±2.07	106.95±3.10	114.57±2.07
	1	80.26±0.10	59.99±0.07	80.26±0.10
	2	36.94±0.12	11.33±0.01	34.94±0.12
	3	8.93±0.12	3.29±0.12	5.93±0.12
	4	3.68±0.01	1.02±0.12	1.68±0.01
	5	3.62±0.12	0.91±0.01	0.92±0.00
	6	0.99±0.01	0.91±0.00	0.92±0.00

Table S4. Content of metabolic compounds produced at the end of fermentation

Compound	Concentration (mg/L)	Concentration (mg/L)	Concentration (mg/L)	Concentration (mg/L)	Concentration (mg/L)	Concentration (mg/L)	Concentration (mg/L)
	**1:1**	**50:1**	**100:1**	**500:1**	**1000:1**	***S.pombe***	***WY-1***
**Esters**							
ethyl acetate	18.40±0.07 a	17.41±0.06 bc	16.92±0.10 c	11.42±0.16 d	9.83±0.23 e	10.20±0.00	19.12±0.00
Isoamyl acetate	1.84±0.08 ab	1.94±0.26 ab	2.93±0.59 a	1.00±0.06 b	0.41±0.18 b	0.43±0.00	2.02±0.00
Ethyl lactate	6.85±0.22 a	4.60±0.36 a	9.12±0.74 a	5.31±0.06 a	5.23±0.05 a	6.26±0.00	11.44±0.00
**Aldehydes**							
acetaldehyde	40.05±0.78 a	36.26±1.71 ab	29.12±1.77 b	35.70±1.98 ab	33.41±0.23 ab	34.33±0.00	37.03±0.00
acetal	3.12±0.26 a	2.92±0.34 ab	2.87±0.00 ab	2.85±0.01 ab	2.85±0.02 ab	1.48±0.00	5.87±0.00
**Alcohols**							
1-propanol	16.34±0.84 b	18.66±1.99 ab	21.30±0.08 a	21.54±0.69 a	21.77±0.80 a	28.05±0.00	16.14±0.00
Isobutanol	49.43±1.61 a	45.32±1.92 ab	41.65±0.55 b	28.60±0.43 c	10.62±0.94 d	4.99±0.00	45.36±0.00
Isoamyl alcohol	252.88±3.94 a	247.76±3.56 a	165.80±3.50 b	152.56±2.84 b	65.67±1.20 c	29.14±0.00	250.08±0.00
Phenylethanol	26.85±0.25 a	19.66±0.54 c	17.01±0.21 d	15.98±0.82 d	17.07±0.13 d	15.83±0.00	28.71±0.00
	**M 0 h**	**M 24h**	**M 48h**	**M 60h**	**M 72h**	***S.pombe***	
**Esters**							
ethyl acetate	16.04±0.28 a	15.62±0.10 a	12.86±0.71 b	11.16±0.07 b	10.28±0.19 b	10.20±0.00	
Isoamyl acetate	2.24±0.01 a	1.83±0.49 a	0.89±0.07 bc	0.84±0.01 bc	0.66±0.02 c	0.43±0.00	
Ethyl lactate	4.05±0.17 d	4.08±0.18 cd	5.07±0.33 c	8.13±0.05 a	6.15±0.30 b	6.26±0.00	
**Aldehydes**							
acetaldehyde	34.14±0.23 a	33.51±1.31 a	28.36±1.06 a	35.70±0.98 a	33.41±0.23 a	34.33±0.00	
acetal	4.04±0.27 a	2.50±0.25 ab	0.43±0.43 b	0.00±0.00 b	0.00±0.00 b	1.48±0.00	

**Alcohols**						
1-propanol	15.09±0.66 d	18.07±1.15 cd	21.27±0.43 ab	22.44±0.27 ab	23.47±0.16	28.05±0.00
Isobutanol	42.35±1.49 a	47.87±3.23 a	34.43±1.87 b	20.72±0.33 c	6.39±0.04	4.99±0.00
Isoamyl alcohol	256.16±1.89 a	266.02±15.10 a	118.12±5.31 c	89.75±1.10 d	33.36±0.30 a	29.14±0.00
Phenylethanol	20.87±0.71 a	20.76±0.39 a	19.54±1.77 ab	16.52±0.23 bc	15.40±0.32 c	15.83±0.00

	S.pombe	**SP+IO**	**SP+TD**	**SP+PK**	**SP+WA**
**Esters**					
ethyl acetate	60.51±2.14	82.83±3.82 a	41.88±0.98 c	72.68±0.06 b	74.09±0.93 ab
Isoamyl acetate	0.25±0.16	0.00±0.00 b	0.00±0.00 b	0.29±0.00 a	0.24±0.08 b
Ethyl lactate	13.43±0.14	12.66±1.08 a	8.75±0.17 b	11.04±0.00 ab	9.11±0.43 b
**Aldehydes**					
acetaldehyde	46.00±0.55	52.88±1.65 b	32.98±1.08 d	40.49±0.00 c	89.80±0.16 a
acetal	60.30±2.05	81.72±3.68 a	42.22±0.94 c	71.87±0.00 b	68.40±0.23 b
**Alcohols**					
1-propanol	31.90±0.68	25.58±0.98 b	35.68±0.79 a	28.11±0.06 b	27.34±0.18 b
Isobutanol	22.81±0.93	42.20±0.86 a	29.77±0.53 b	24.93±2.69 bc	22.58±0.22 c
Isoamyl alcohol	97.62±1.53	127.95±1.98 b	134.21±0.35 a	100.79±0.00 c	68.23±0.16 d
Phenylethanol	12.98±0.08	13.18±0.06 c	14.66±0.20 a	12.85±0.00 c	13.76±0.06 b

	S.pombe	**SP+TD**	**M:SP+TD**	**M60SP+TD**
**Esters**				
ethyl acetate	55.26±0.00	36.01±0.44 a	33.67±0.88 b	31.35±0.60 b
Ethyl lactate	10.65±0.00	6.92±0.02 bc	6.74±0.17 c	5.92±0.02 d
**Aldehydes**				
acetaldehyde	20.20±0.00	12.95±0.06 c	10.00±0.04 d	9.95±0.06 d

acetal	89.40±0.00	48.12±0.44 d	27.29±0.27 e	47.46±0.24 d
**Alcohols**				
1-propanol	28.13±0.00	33.05±0.01 a	33.83±0.41 a	33.03±0.02 a
Isobutanol	18.32±0.00	34.53±0.14 d	37.84±0.87 bc	35.20±0.75 cd
Isoamyl alcohol	90.55±0.00	149.40±0.33 bc	161.28±0.50 a	159.37±0.37 a
Phenylethanol	15.07±0.00	25.26±0.24 a	24.56±0.17 a	25.26±0.27 a

Table S5. Ethanol content produced during fermentation

Different Inculation Ratios	Concentration (mg/L)	Different Inculation interval	Concentration (mg/L)	Different Co-Fermentation Strategies with Non-Saccharomyces	Concentration (mg/L)	Different Mixed Fermentation Strategies	Concentration (mg/L)
1:1	69.30±0.35	M 0h	67.18±0.01	SP＋IO	61.59±2.10	SP＋TD	75.39±0.01
50:1	68.18±0.41	M 24h	69.22±0.52	SP＋TD	61.60±1.10	M:SP＋TD	76.26±0.00
100:1	68.88±0.20	M 48h	74.23±0.41	SP＋PK	63.51±1.10	M60SP＋TD	75.39±0.01
500:1	69.96±0.10	M 60h	74.39±0.01	SP＋WA	62.14±0.00		
1000:1	69.12±0.11	M 72h	75.45±0.01				
S.pombe	74.12±0.01						
WY-1	70.00±0.20						

Table S6. Glycerol content produced during fermentation

Different Inculation Ratios	Concentration (mg/L)	Different Inculation interval	Concentration (mg/L)	Different Co-Fermentation Strategies with Non-Saccharomyces	Concentration (mg/L)	Different Mixed Fermentation Strategies	Concentration (mg/L)
1:1	5.06±0.02	M 0h	5.16±0.01	SP＋IO	5.57±0.08	*S.pombe*	6.42±0.14
50:1	5.32±0.02	M 24h	5.24±0.18	SP＋TD	5.50±0.07	SP＋TD	5.92±0.12
100:1	5.62±0.11	M 48h	6.23±0.23	SP＋PK	5.35±0.10	M:SP＋TD	5.27±0.12
500:1	6.66±0.09	M 60h	6.55±0.35	SP＋WA	5.78±0.01	M60SP＋TD	5.76±0.12
1000:1	7.29±0.11	M 72h	6.68±0.24				
S.pombe	6.98±0.19						
WY-1	5.39±0.01						

Table S7. Colour values of the finishing wines

Different Inoculation Ratios	Chromaticity Value	Different Inoculation Interval	Chromaticity Value	Different Co-Fermentation Strategies with Non-Saccharomyces	Chromaticity Value	Different Mixed Fermentation Strategies	Chromaticity Value
1:1	0.68±0.03 a	M0	0.63±0.00 b	SP＋IO	1.69±0.07 c	*WY-1*	1.89±0.07 b
50:1	0.70±0.06 a	M24	0.56±0.02 b	SP＋TD	2.02±0.03 a	*S.pombe*	2.38±0.07 a
100:1	0.67±0.09 a	M48	0.77±0.08 b	SP＋PK	1.82±0.02 b	SP＋TD	2.02±0.03 a
500:1	0.73±0.02 a	M60	1.01±0.08 a	SP＋WA	1.39±0.01 d	M:SP＋TD	1.33±0.01 c
1000:1	0.95±0.03 a	M72	1.12±0.04 a			M60SP＋TD	1.92±0.03 b

Table S8. Descriptive sensory evaluation of the finishing wines

	S.pombe score	**SP＋TD** score	**M:SP＋TD** score	**M60SP＋TD** score
Colour	5.78±0.40 a	5.44±0.30 a	4.44±0.30 a	4.56±0.55 a
Nail polish aroma	4.10±0.10 a	4.05±0.05 a	3.11±0.11 b	3.03±0.03 b
Almond flavor	5.56±0.30 a	5.11±0.01 a	5.12±0.12 a	5.11±0.11 a
Pear flavor	2.15±0.15 a	2.30±0.30 a	2.26±0.26 a	2.62±0.31 a
Grassy notes	1.03±0.03 a	1.04±0.04 a	1.20±0.20 a	1.20±0.20 a
Banana flavor	2.07±0.07 a	2.04±0.04 a	2.08±0.08 a	2.07±0.07 a
Rose scent	6.08±0.08 a	6.08±0.08 a	6.08±0.08 a	6.22±0.22 a
Sour taste	2.04±0.04 b	2.70±0.35 b	4.22±0.22 a	4.08±0.08 a
